# Quantitative analysis of SPECT imaging parameters in patients with resting perfusion defects on myocardial perfusion scintigraphy

**DOI:** 10.4103/0972-3919.78245

**Published:** 2010

**Authors:** Ankur Pruthi, Ramesh Asopa, MGR Rajan, Sandip Basu

**Affiliations:** Radiation Medicine Centre, Bhabha Atomic Research Centre (BARC), Tata memorial hospital (Annexe Bldg.), Mumbai, India

**Keywords:** Emory cardiac toolbox, hibernating myocardium, left ventricular failure, myocardial perfusion imaging, myocardial viability, quantitative parameters

## Abstract

**Background:**

Functional status/contractile behaviour of hibernating myocardium was analyzed objectively by analyzing the available quantitative parameters obtained on gated SPECT myocardial perfusion imaging (MPI) using Emory cardiac toolbox (ECTB) software.

**Materials and Methods:**

In this retrospective study, 70 patients with perfusion defects on ^99^Tc-Sestamibi MPI (12 females, 58 males) who also underwent ^18^F-FDG Cardiac PET study for assessment of hibernating myocardium were included for analysis. Patients were divided in three categories based on summed rest score (SRS) obtained from ECTB software, depicting the extent of perfusion defects. In a study population matched for extent of perfusion defects, quantitative parameters obtained from ECTB software such as left ventricular ejection fraction (LVEF), left ventricular end diastolic volume (EDV), left ventricular end systolic volume (ESV) and left ventricular stroke volume (SV) were compared between patients showing evidence of hibernating myocardium and patients showing no evidence of hibernating myocardium. Student ‘t’ test was applied on the given observations and a *P*-value <0.05 was considered as a significant difference between the means in two categories.

**Results:**

There was no significant difference in LVEF, EDV, ESV and SV measurements between those who demonstrate hibernating myocardium and those who show no evidence of hibernating myocardium across all the categories of patients. Few trends were evident in the present study in LVEF, EDV and ESV measurements i.e., fall in mean LVEF with increasing SRS and rise in mean EDV and ESV with increasing SRS.

**Conclusions:**

The findings were consistent with the nature of hibernating myocardium i.e., non-contractile and dysfunctional. The fall in the LVEF was suggestive of deteriorating myocardial function with increasing extent of perfusion defects. The increasing left ventricular EDV and ESV with increasing extent of perfusion defects was suggestive of rising incidence of gross morphological LV cavity dilatation or “Dilated ischemic cardiomyopathy” in these patients.

## INTRODUCTION

Hibernating myocardium is a state of persistently impaired myocardial function due to reduced coronary blood flow that can be partially or completely restored to normal if the myocardial oxygen supply/demand relationship is favorably altered, either by improving blood flow and/or by reducing demand.[1,6,7]

The concept of hibernation presupposed that a reduction in coronary blood flow was followed by a down regulation in cardiac function to a point at which the limited oxygen supply enabled the maintenance of the biochemical functions that sustained cell integrity.[1] The hibernating response of the heart is considered as an act of self-preservation.[1]

The primarily clinical concept of myocardial hibernation subsequently merged with a number of experimental observations: (a) Regional myocardial function and blood flow are reduced proportionately during ischemia i.e., a state of perfusion-contraction matching (b) Metabolic parameters such as myocardial lactate consumption, creatine phosphate content and free energy change of ATP-hydrolysis recover toward their preischemic baseline values during ongoing ischemia, consistent with the idea that the reduced function is an adaptation to reduced blood flow. (c) An inotropic reserve persists in hibernating myocardium.[3]

The phenotype of hibernating myocardium, i.e., chronic, yet reversible contractile dysfunction in the setting of coronary artery disease could arise from either continued ischemia or from repetitive cycles of ischemia/reperfusion.[3] Hibernating myocardium refers to the presence of persistent myocardial and left ventricular dysfunction at rest, associated with conditions of severely reduced coronary blood flow.[2]

Several non-invasive techniques have been developed to identify viable myocardium in the dysfunctional segments:[5]

- ^18^F-FDG cardiac PET.
- ^99m^Tc-Sestamibi.
- ^99m^Tc-Tetrofosmin.
- ^201^Tl rest-redistribution technique.
- ^201^Tl reinjection technique.
- Low dose dobutamine stress echocardiography.

Cardiac ^18^F-FDG PET is the “most sensitive” marker for hibernating myocardium while Dobutamine stress echocardiography is considered to be the “most specific” marker.[5]

We sought to assess the contractile/functional behavior of hibernating myocardium by comparing quantitative parameters like left ventricular ejection fraction (LVEF), left ventricular end diastolic volume (EDV), left ventricular end systolic volumes (ESV) and left ventricular stroke volume (SV) [obtained from myocardial perfusion imaging (MPI) studies] between individuals having evidence of hibernating myocardium and individuals with no evidence of hibernating myocardium.

## MATERIALS AND METHODS

A retrospective analysis was carried out on 70 patients with perfusion defects on ^99m^Tc-Sestamibi MPI [Table 1] who also underwent ^18^F-FDG cardiac PET study for the assessment of hibernating myocardium. Assessment was done visually by a single nuclear medicine physician and “Perfusion-metabolism mismatch” in any one segment on visual analysis was taken as the criterion for diagnosing hibernating myocardium.[6]

**Table 1:** Patient characteristics used in the study

Total patients	70
Sex	
Males	58
Females	12
History of myocardial infarction	54
No history of myocardial infarction	14

Patients were divided in three categories on the basis of summed rest score (SRS) depicting the extent of defects:

- SRS between 0 and 10.
- SRS between 11 and 20.
- SRS between 21 and 30.

**Figure 1:**
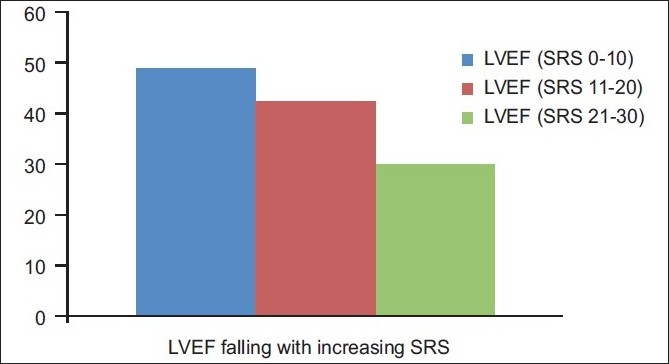
The graph showing fall in left ventricular ejection fraction with increasing summed rest score

**Figure 2:**
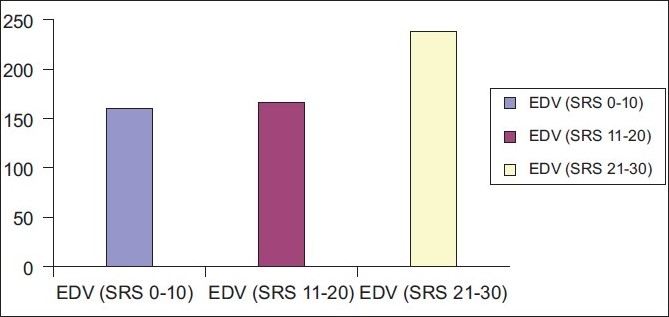
The graph showing rising left ventricular end diastolic volume with rising summed rest score

**Figure 3:**
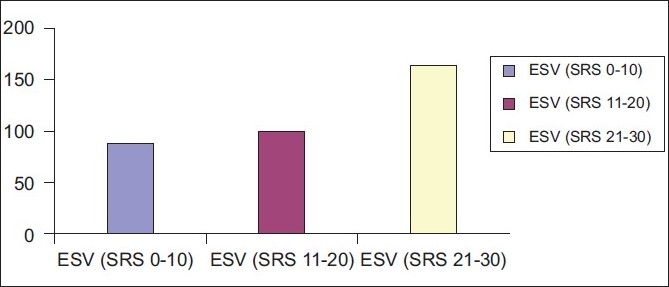
The graph showing rising left ventricular end systolic volume with increasing summed rest score

In this study population matched for the extent of defects, quantitative parameters such as LVEF, EDV, ESV and SV obtained from Emory Cardiac toolbox (ECTB) software were compared between patients showing evidence of hibernating myocardium and patients showing no evidence of hibernating myocardium [Figures 1–3].

### Inclusion criteria

All left ventricular failure patients irrespective of prior myocardial infarction, who underwent Tc-^99m^ Sestamibi (hexakis methoxy isobutyl isonitrile) MPI and 18- FDG (Fluoro deoxy glucose) cardiac PET study for assessment of hibernating myocardium (from January 2008 to March 2010 at our institute) were included in the study.

### Imaging protocol

Rest myocardial perfusion scintigraphy was performed 1 hour following intravenous administration of ^99m^Tc-Sestamibi. Images were obtained on Siemens dual head γ-camera (E cam) by Gated SPECT in supine position from RAO 45°to LPO 225°. The images were reconstructed into vertical long, horizontal long and short-axis slices. Quantitative parameters such as LVEF in percentage, EDV in ml, ESV in ml, SV in ml and SRS were obtained along with a polar map depicting “17 segment model” using ECTB software using a semi-automatic approach. The role of operator was limited to defining correctly the left ventricular endocardial and epicardial borders.

On another day (within 3 days of the MPI study), 90 minutes after intravenous injection of ^18^F-FDG, which was administered 1 hour after oral glucose load of 75 g, a regional cardiac PET study was acquired using a whole-body full-ring dedicated GE Advance BGO PET camera. Images acquired were processed into short axis, vertical long axis and horizontal long axis. A polar map depicting “17 segment model” was also generated.

### Analysis

Assessment of hibernating myocardium was done visually by single physician. “Perfusion-metabolism mismatch” in any one segment on visual analysis was taken as the sole crieterion for hibernating myocardium.

### Statistical analysis

Mean values and standard deviations of the quantitative parameters in different categories were calculated. Unpaired “Student ‘*t*’ test” was applied to the data in each category for each parameter and *P*-value <0.05 was considered as significant difference between the mean values.

## RESULTS

Results are shown in Tables 2–5

**Table 2:** Combined results of all patients

	Group with evidence of hibernating myocardium (39 patients)	Group with no evidence of hibernating myocardium (31 patients)	*P*-value (obtained on student ‘*t*’ test)
Mean LVEF (%)	39.10 ± 16.75	41.35 ± 16.61	0.5767
Mean EDV (ml)	173.71 ± 74.57	186.71 ± 111.86	0.562999
Mean ESV (ml)	113.67 ± 67.74	121.90 ± 102.05	0.687087
Mean SV (ml)	60.05 ± 22.77	64.81 ± 17.77	0.3435744

LVEF: left ventricular ejection fraction; EDV: left ventricular end diastolic volume; ESV: left ventricular end systolic volume; SV: left ventricular stroke volume (Mean ± Standard Deviation)

**Table 3:** Results for the category “SRS 0-10”

	Group with evidence of hibernating myocardium (7 patients)	Group with no evidence of hibernating myocardium (8 patients)	*P*-value (obtained on student ‘*t*’ test)
Mean LVEF (%)	42.57 ± 19.06	55.12 ± 14.79	0.1748
Mean EDV (ml)	188.14 ± 75.67	121.37 ± 52.11	0.065251
Mean ESV (ml)	118.85 ± 79.61	60 ± 36.93	0.082737
Mean SV (ml)	69.28 ± 24.44	61.37 ± 17.78	0.4820809

LVEF: left ventricular ejection fraction; EDV: left ventricular end diastolic volume; ESV: left ventricular end systolic volume; SV: left ventricular stroke volume (Mean ± Standard Deviation)

**Table 4:** Results for the category “SRS 11-20”

	Group with evidence of hibernating myocardium (26 patients)	Group with no evidence of hibernating myocardium (14 patients)	*P*-value (obtained on student ‘*t*’ test)
Mean LVEF (%)	40.61 ± 17.10	43.35 ± 14.40	0.6133
Mean EDV (ml)	154.65 ± 61.68	169.35 ± 63.54	0.481003
Mean ESV (ml)	98.34 ± 55.89	102.85 ± 53.78	0.80655
Mean SV (ml)	56.31 ± 21.97	66.5 ± 14.02	0.1253484

LVEF: left ventricular ejection fraction; EDV: left ventricular end diastolic volume; ESV: left ventricular end systolic volume; SV: left ventricular stroke volume (Mean ± Standard Deviation)

**Table 5:** Results for the category “SRS between 21-30”

	Group with evidence of hibernating myocardium (5 patients)	Group with no evidence of hibernating myocardium (10 patients)	*P*-value (obtained on student ‘*t*’ test)
Mean LVEF (%)	30.6 ± 7.30	29.9 ± 10.35	0.8953
Mean EDV (ml)	252.2 ± 100.21	209.4 ± 55.92	0.300641
Mean ESV (ml)	180 ± 85.20	149.9 ± 60.84	0.441727
Mean SV (ml)	72.2 ± 19.84	59.5 ± 15.34	0.1921704

LVEF: left ventricular ejection fraction; EDV: left ventricular end diastolic volume; ESV: left ventricular end systolic volume; SV: left ventricular stroke volume (Mean ± Standard Deviation)

## DISCUSSION

To summarize the overall results, the following observations were made:

There was no significant difference in LVEF, EDV, ESV and SV measurements between those who demonstrate hibernating myocardium and those who show no evidence of hibernating myocardium across all the categories of patients.

Few trends were evident in the present study in LVEF, EDV and ESV measurements:

- Fall in mean LVEF with increasing SRS.
- Mean EDV and ESV rising with increasing SRS.

In review of the literature, Schinkel *et al*, and Rahimtoola *et al*, have described hibernating myocardium as non-contractile and dysfunctional.[1,7] Our study is in concordance with the known description of hibernating myocardium and is an indirect and objective reference of its non-contractile behavior. Phenotypically, hibernating myocardium was found to behave similar to infracted myocardium in terms of LVEF and left ventricular volumes. Our study was able to assess the contractile behavior of hibernating myocardium without the use of 2D-echocardiograpy.

LVEF was found to be inversely proportional to the SRS i.e., systolic dysfunction was found to increase with increase in extent of perfusion defect.

In their study Carluccio E *et al*,[8] have presented thatin patients with either non–Q-wave MI or no previous MIbut with LV wall motion abnormality and hibernating myocardium, there is remodellingof the LV i.e., LV EDV and ESV are increased and the LV is more spherical. Thus, they have documented that the mere presence of LV systolic dysfunctionwith hibernating myocardium can lead to LV remodelling. They have also documentedthat revascularization in patients with hibernating myocardium results in reverseremodelling i.e., there is a reduction of the increased LVEDVand LVESV, the LV is less spherical and LVEF increases.

Findings of our study are in agreement with the above study. Our study shows increment in left ventricular systolic and diastolic volumes or left ventricular cavity dilatation with increase in extent of perfusion defects.

## CONCLUSIONS

The quantitative parameters obtained didn’t show any significant difference in any of the categories of patients. These findings were consistent with the nature of hibernating myocardium i.e., non-contractile and dysfunctional myocardium.

The trends in the LVEF, EDV and ESV were suggestive of deteriorating myocardial function with increasing extent of perfusion defects.

The increase in left ventricular EDV and ESV with increasing extent of perfusion defects was suggestive of increasing incidence of gross morphological LV cavity dilatation or “Dilated ischemic cardiomyopathy” in these patients.
